# Voluntary Medical Male Circumcision: An Introduction to the Cost, Impact, and Challenges of Accelerated Scaling Up

**DOI:** 10.1371/journal.pmed.1001127

**Published:** 2011-11-29

**Authors:** Catherine Hankins, Steven Forsythe, Emmanuel Njeuhmeli

**Affiliations:** 1 Joint United Nations Programme on HIV/AIDS, Geneva, Switzerland; 2 Futures Institute, Glastonbury, Connecticut, United States of America; 3 United States Agency for International Development, Washington, District of Columbia, United States of America; Centers for Disease Control and Prevention, United States of America

## Abstract

Scaling up voluntary medical male circumcision (VMMC) for HIV prevention is cost saving and creates fiscal space in the future that otherwise would have been encumbered by antiretroviral treatment costs. An investment of US$1,500,000,000 between 2011 and 2015 to achieve 80% coverage in 13 priority countries in southern and eastern Africa will result in net savings of US$16,500,000,000. Strong political leadership, country ownership, and stakeholder engagement, along with effective demand creation, community mobilisation, and human resource deployment, are essential. This collection of articles on determining the cost and impact of VMMC for HIV prevention signposts the way forward to scaling up VMMC service delivery safely and efficiently to reap individual- and population-level benefits.

## Abstract 2

Catherine Hankins, Steven Forsythe, and Emmanuel Njeuhmeli provide an overview of the “Voluntary Medical Male Circumcision for HIV Prevention: The Cost, Impact, and Challenges of Accelerated Scale-Up in Southern and Eastern Africa” Collection, calling for leadership and vision to help halt the HIV epidemic.

## Male Circumcision for HIV Prevention

Despite a 22-fold increase since 2001 in the number of people receiving antiretroviral therapy, two people acquire HIV infection for every person starting treatment [1]. Scaling up evidence-informed HIV prevention programs is imperative. In the HIV prevention toolbox of behavioural, biomedical, and structural approaches to combine for maximum effect [2], VMMC is an essential tool in all high HIV prevalence, predominantly heterosexual epidemic settings. It provides lifelong partial protection for men against HIV infection [3]–[6] and reduces their likelihood of genital ulcers [7],[8], syphilis [9], and penile cancer [10].

Observational data and ecological studies have suggested for decades that male circumcision provides a level of protection from HIV infection for men [11]. Three randomised controlled trials [3]–[5] conducted in the last decade found a 57% protective effect against HIV for men who became circumcised [6]. All three trials were stopped prematurely because it was deemed unethical to withhold VMMC from men in the control arm waiting to be circumcised.

Following trial results, the World Health Organization (WHO) and the Joint United Nations Programme on HIV/AIDS (UNAIDS) rapidly convened stakeholders in March 2007 to evaluate the strength of the evidence and to consider the policy and programmatic implications. The resulting recommendations addressed the essential components for program implementation in 13 priority countries (Botswana, Kenya, Lesotho, Malawi, Mozambique, Namibia, Rwanda, South Africa, Swaziland, Tanzania, Uganda, Zambia, and Zimbabwe) in eastern and southern Africa with settings of high HIV prevalence and low levels of male circumcision [12],[13].

Male circumcision is the oldest and most common surgical procedure. With 30% of men globally and 67% of men in sub-Saharan Africa circumcised [14], social and cultural factors are the main determinants of acceptability [6],[15]–[18]. In sub-Saharan Africa, male circumcision was found to be acceptable to men and women in non-circumcising communities if readily accessible and provided safely [18]. Mathematical modelling has shown that medical male circumcision is highly cost-effective, with costs to avert one HIV infection ranging from US$150 to US$900 using a ten-year time horizon, and one new HIV infection averted for every five to fifteen procedures performed [19].

Given these levels of acceptability, cost, and potential impact, VMMC provided by well-trained, well-equipped providers in hygienic settings should be scaled up rapidly in high HIV prevalence settings to reap both individual- and population-level benefits. Policy makers and program planners interested in moving services to scale are faced, however, with decisions about which populations to prioritise (newborns, adolescents, adults, men at higher risk of HIV exposure, such as those in serodiscordant couples), what service delivery models to use (fixed, outreach, mobile), which human resources to deploy (physicians, clinical officers, nurses), how to create demand and match supply to it, and what speed of scale-up is both desirable and feasible.

Policy makers in high HIV prevalence countries will find valuable information to support rapid scale-up of services in this collection on the cost and impact of VMMC for HIV prevention. The articles highlight progress to date, explore challenges to overcome, and provide solutions to facilitate scale-up. By moving forward now, leaders will set their countries on course to achieve the 50% reduction in sexual transmission of HIV by 2015 that they signed on to at the United Nations General Assembly in June 2011 [20].

## Numbers of Male Circumcisions Needed

How many medical male circumcisions will need to be performed in the 13 priority countries for maximum impact? The first step towards answering this question is assessing baseline prevalence of male circumcision in each country, recognising that self-reported circumcision status, as Thomas et al. [21] demonstrate for Lesotho, can be inaccurate. Njeuhmeli et al. [22] estimate the number of VMMC procedures needed to reach 80% prevalence using the Decision Makers' Program Planning Tool [23] for male circumcision, an interactive tool that incorporates country-specific demographics, epidemic dynamics, and locally derived cost estimates. An estimated 20.3 million circumcisions among men 15–49 years of age are needed to close the gap by 2015. Once eligible uncircumcised men have been reached through “catch-up” campaigns, annual costs fall because ongoing programs need only maintain coverage while shifting to long-term sustainable strategies. These could include systematically offering the procedure to one cohort per year, such as young men turning 15 years of age or all male newborns.

**Figure 1:**
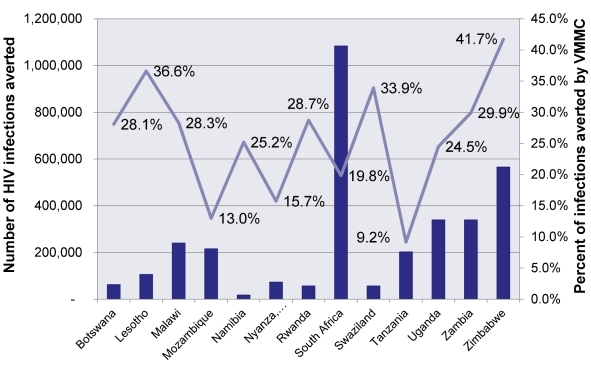
Achievement towards target of 80% coverage. This figure illustrates that most countries have had only limited success in bridging the gap between historical male circumcision levels and the 80% target. The one exception is Kenya, which has achieved more than 66% of its objective, primarily in Nyanza Province.

As Mwandi et al. [24] detail, Kenya is on track, with over 66% of its target for Nyanza Province met through sustained government leadership, financial and technical support of international donors, and a partnership strategy that has engaged a broad array of stakeholders. Swaziland has reached 13%, Zambia and Botswana have attained 4%, and Tanzania and South Africa have met 3% of their respective 2015 80% objectives, through campaigns that picked up speed in 2010 (see Figure 1).

## Efficiently Mobilising Human Resources

Among the impediments to scaling up VMMC services are concerns about human resources. Curran et al. [25] review the concepts of task shifting and task sharing and describe approaches to expanding the health sector workforce through redeployment of existing personnel and use of expatriate volunteers, drawing on experiences in Tanzania, Kenya, and Swaziland. Scaling up VMMC requires a streamlined campaign footing and rapid mobilisation of human resources to eventually achieve a steady state that will make fewer demands on the health care system. Both surgical and non-surgical efficiency gains have significant impacts on per procedure costs and therefore on the numbers of medical male circumcisions that can be performed. Significant efforts are underway to test a number of medical devices that, if found to be safe, acceptable, and effective, would dramatically reduce procedure times [26]–[28] and speed the scale-up.

## Determining Costs of Scale-Up

VMMC scale-up planning requires cost estimates for commodity procurement, supply chain management, and disposal of waste generated by male circumcision programs. Edgil et al. [29] describe a costing framework for these program components for Swaziland, emphasising the advantages of a standard kit of the consumables and dedicated instruments necessary for one male circumcision procedure [30],[31].

VMMC program planning necessitates the design and implementation of effective demand creation strategies that encourage men to consider male circumcision for HIV prevention and actively seek out VMMC services. Timely matching of supply to demand is critical to avoid men seeking unsafe procedures because waiting times are too long. Mahler et al. [32] describe how this challenge was met while ensuring service quality and efficiency in Iringa, Tanzania. As Bertrand et al. [33] underscore, demand creation strategies must be tailored to specific country contexts to determine the most effective mix of mass media, small media, outreach, and community mobilisation communication approaches. Recognising that no standard package applies in all settings, they propose a seven-step methodology to estimate demand creation costs for inclusion in country VMMC costing estimates.

The United States President's Emergency Plan for AIDS Relief (PEPFAR) (through the US Agency for International Development Health Policy Initiative) and the UNAIDS supported primary data collection activities to estimate facility-based unit costs for VMMC, as currently delivered, in Uganda, Kenya, Zambia, Zimbabwe, South Africa, and Namibia. These estimates were revised to include a more comprehensive assessment of cost components, including waste management, supply chain management, training, and overhead costs. Assumptions were made about future task shifting and task sharing to optimise the volume and efficiency of services [31]. Unit cost estimates, excluding demand creation costs, were estimated at US$80.13 per adult VMMC. Country unit costs were then adjusted to account for differences in labour costs, and sensitivity analyses assumed a 20% higher and 20% lower unit cost.

Combining unit cost data with the numbers of VMMCs needed to achieve 80% coverage in all 13 countries results in an estimated US$1,500,000,000 required between 2011 and 2015. Maintaining 80% coverage in all 13 countries between 2016 and 2025 would require an additional US$500,000,000. Sensitivity analyses underscore the importance of minimising costs while ensuring safe, high-quality procedures accompanied by effective HIV prevention messages. If unit costs were 20% higher, an additional US$500,000,000 would be required over the full 2011–2025 period, while a 20% decrease in unit costs would reduce overall resources required by US$500,000,000. Given the estimated discounted individual lifetime cost of antiretroviral therapy of US$7,400, VMMC is not only cost-effective in these countries—it is cost saving. Net savings from 2011 to 2015 due to averted treatment and care costs amount to US$16,500,000,000. Therefore, an initial investment now in VMMC, although substantial, will be returned many fold. It will create fiscal space in the future that otherwise would have been encumbered by antiretroviral treatment costs.

## Estimating the Impact

**Figure 2:**
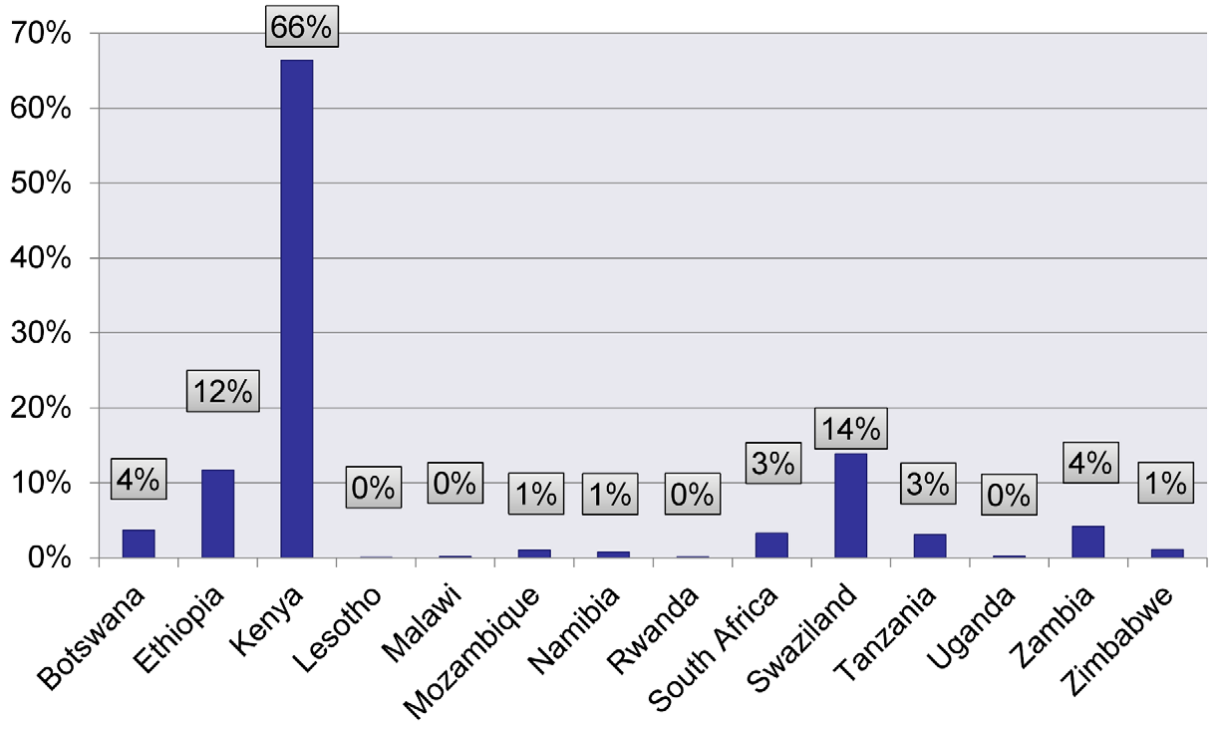
Cumulative number and percentage of HIV infections averted between 2011 and 2025 by scaling up adult VMMC to reach 80% coverage in five years. This figure illustrates the significant impact that achieving 80% VMMC coverage of 15- to 49-year-old men would have on the epidemics in 13 countries in eastern and southern Africa. South Africa can avert the largest number of HIV infections (over 1 million between 2011 and 2025); Zimbabwe can avert the highest percentage of new HIV infections (almost 42%). More than 20% of new HIV infections would be averted between 2011 and 2025 in nine countries: Botswana, Lesotho, Malawi, Namibia, Rwanda, Swaziland, Uganda, Zambia, and Zimbabwe. Nyanza refers to Nyanza Province in Kenya: the data presented are only for Nyanza Province in Kenya, as this is the only province in Kenya with prevalence of male circumcision lower than 80% and is the province with the highest HIV prevalence compared to the national average.

Just how many infections could be averted by scaling up VMMC to reach 80% male circumcision prevalence in all countries in eastern and southern Africa in five years and maintaining that coverage through to 2025? Where would the largest impact be seen? Through 2025, 3.4 million new HIV infections would be averted, as Figure 2 shows, with South Africa alone averting over 1 million new HIV infections between 2011 and 2025. More than 20% of projected new HIV infections would be averted in Botswana, Lesotho, Malawi, Namibia, Rwanda, Swaziland, Uganda, Zambia, and Zimbabwe. The number of VMMCs needed to avert one HIV infection ranges from a low of four in Zimbabwe to a high of 44 in Rwanda.

Among the infections averted are those among women who benefit indirectly from VMMC scale-up. As more men become circumcised, women are less likely to encounter sexual partners who have HIV infection. Eventually, even uncircumcised men will benefit indirectly from VMMC scale-up [19]. Early on, most HIV infections averted occur among men, but the proportion among women steadily increases over time until almost half of all HIV infections averted in the year 2025 are those that would have occurred among women.

The anticipated impact of scaling up VMMC is directly proportional to the pace and scale of implementation. Decreasing VMMC coverage targets from 80% to 50% results in a decline in the total number of HIV infections averted from 3.4 million to 1.1 million. Reducing the time to achieve 80% coverage from five years to one year has the opposite effect, increasing the number of HIV infections averted from 3.4 million to 4.1 million. This decreases the cost per HIV infection averted and increases total cost savings due to HIV infections averted. Maximum epidemic impact accrues the more quickly maximum coverage is achieved.

## Determinants of Early Adoption and Sustained Scale-Up

Some countries need to offer medical male circumcision to fewer than 500,000 men and could achieve 80% coverage in less than two years, while those that have to offer circumcision to more than 2–3 million men will take longer. However, it is not just the size of the task that will determine whether objectives are met, impact is realised, and cost savings occur. As Dickson et al. [34] demonstrate, key barriers and facilitators are influencing the speed of scale-up. Characteristics of the response to the compelling scientific evidence of the HIV prevention benefits of VMMC in each of the 13 countries permit a classification of countries into four categories: innovators, early adopters, early and late majority, and laggards. Against a backdrop of varying sociopolitical and cultural contexts, the key drivers of early adoption and sustained scale-up are country ownership, explicit political leadership, engagement of stakeholders, and community mobilisation.

## Conclusion

What will it take for the citizens of the 13 priority countries in eastern and southern Africa to reap the prevention benefits of VMMC? Domestic funding can be mobilised and international funding accessed through PEPFAR, the Bill & Melinda Gates Foundation, and the Global Fund to Fight AIDS, Tuberculosis and Malaria. However, it will take leadership and visible champions at all levels to mobilise and deploy this funding for maximum effect. This is precisely the “decisive, inclusive, and accountable leadership” called for in the United Nations' 2011 political declaration on HIV/AIDS [20]. It will take community conversations to create new social norms about male circumcision in previously non-circumcising communities. It will take women speaking out on the HIV prevention benefits and the desirability of male circumcision for their sexual partners, brothers, and sons. It will take tailored communication strategies to create demand for VMCC services, and it will take program planners who have anticipated increasing demand and are matching it with supply of safe, acceptable, and accessible services. It will take continued innovations in medical device development and testing, along with other efficiency gains, to decrease procedure times and thereby increase access.

Above all, it will take vision by government leaders who understand that promoting effective VMMC programming now will create synergies to more rapidly halt and reverse their countries' epidemics. Scaling up VMMC requires considerable short-term investment of financial and human resources to accelerate gains toward coverage objectives. Once achieved, countries will arrive at a sustained and sustainable “cruising” level requiring far fewer resources to maintain 80% coverage. The challenge before them is to climb over the “catch-up” hump to reap substantive HIV prevention benefits. The faster that countries do this, the more rapidly will both direct benefits for men and indirect benefits for women accrue. Six million people are on antiretroviral therapy, 9 million more are eligible today, according to the current WHO recommendations of providing antiretroviral treatment for patients with CD4 cell counts of <350 cells/µl [35], and pressure is mounting to offer even more people treatment for prevention [1],[36]. The opportunity costs of not taking action to scale up VMMC safely and rapidly now to prevent new HIV infections and create fiscal space are too high to ignore. This is on our watch—what role is each one of us playing to ensure that VMMC contributes fully to halting and reversing the HIV epidemic in eastern and southern Africa?

## Abbreviations

PEPFAR: United States President's Emergency Plan for AIDS Relief
UNAIDS: Joint United Nations Programme on HIV/AIDS
VMMC: voluntary medical male circumcision
WHO: World Health Organization
